# Potential Epigenetic-Based Therapeutic Targets for Glioma

**DOI:** 10.3389/fnmol.2018.00408

**Published:** 2018-11-15

**Authors:** Lanlan Zang, Shukkoor Muhammed Kondengaden, Fengyuan Che, Lijuan Wang, Xueyuan Heng

**Affiliations:** ^1^Central Laboratory and Key Laboratory of Neurophysiology, Linyi People’s Hospital, Shandong University, Linyi, China; ^2^Department of Medicinal Chemistry, Key Laboratory of Chemical Biology (Ministry of Education), School of Pharmaceutical Sciences, Shandong University, Jinan, China; ^3^Chemistry Department and Center for Diagnostics and Therapeutics, Georgia State University, Atlanta, GA, United States; ^4^Department of Neurology, Linyi People’s Hospital, Shandong University, Linyi, China

**Keywords:** glioma, epigenetics, DNA methylation, miRNA, chromatin remodeling, histone modifications

## Abstract

Glioma is characterized by a high recurrence rate, short survival times, high rates of mortality and treatment difficulties. Surgery, chemotherapy and radiation (RT) are the standard treatments, but outcomes rarely improve even after treatment. With the advancement of molecular pathology, recent studies have found that the development of glioma is closely related to various epigenetic phenomena, including DNA methylation, abnormal microRNA (miRNA), chromatin remodeling and histone modifications. Owing to the reversibility of epigenetic modifications, the proteins and genes that regulate these changes have become new targets in the treatment of glioma. In this review, we present a summary of the potential therapeutic targets of glioma and related effective treating drugs from the four aspects mentioned above. We further illustrate how epigenetic mechanisms dynamically regulate the pathogenesis and discuss the challenges of glioma treatment. Currently, among the epigenetic treatments, DNA methyltransferase (DNMT) inhibitors and histone deacetylase inhibitors (HDACIs) can be used for the treatment of tumors, either individually or in combination. In the treatment of glioma, only HDACIs remain a good option and they provide new directions for the treatment. Due to the complicated pathogenesis of glioma, epigenetic applications to glioma clinical treatment are still limited.

## Introduction

Glioma is the most common form of primary malignant brain tumors, accounting for nearly 30% of all brain tumors, and also one of the most lethal (Chien et al., 2016). Some symptoms of glioma may be subtle and gradually worsen, while others may be present as an acute illness. The exact mechanism of occurrence of glioma remains unclear.

Recently, the World Health Organization (WHO) Classification of Tumors of the Central Nervous System (CNS; Wen and Huse, 2017) categorized glioma into four grades. Grades I and II are considered low-grade glioma, whereas grades III and IV are high-grades, with the degree of malignancy increasing in the higher grades. The types of glioma include low-grade glioma (WHO II: mainly refers to diffuse astrocytoma, oligodendroglioma, and oligodendrocyte astrocytoma), anaplastic glioma (WHO III), glioblastoma (GBM, WHO IV), brain gliomatosis (pathologically astrocytoma-based, can be divided into WHO II, III, IV) and ependymomas (WHO II, III). Each grade has a relatively specific prognosis to guide the clinical treatment, but most of the glioma are WHO III grade anaplastic glioma and WHO grade IV GBM, which is devoid of cure with the current therapeutic options.

Unfortunately, most high-grade gliomas do not have a definitive cure. The current treatment for these high-grade tumors mainly focuses on surgical resection, followed by chemotherapy and radiotherapy. Temozolomide (TMZ) can traverse the blood-brain barrier and it is often used to treat these tumors. However, various side effects and adverse prognosis exist, such as the hematological toxicity (Stupp et al., 2005), and glioma have proven to be particularly resistant to radiotherapy and chemotherapy. The main mechanism of drug resistance is correlated with the O6-methyl guanine-DNA methyltransferase (MGMT; Hegi et al., 2005), which can bind to an alkyl compound on the O6 of DNA guanine and reduce it so that protein complexes cannot recognize the mismatched base pair, resulting in cell tolerance to O6 guanine methylation. As a result, the efficacy of these treatments is not promising and the prognosis of patients remains poor.

Therapies aimed to overcome these limitations have been presented in recent preclinical and clinical studies, including targeted molecular therapy, immunotherapy, gene therapy and stem cell therapy. The success of targeted molecular therapy for some tumor types, such as non-small cell lung cancer (Antonelli et al., 2016), malignant melanoma (Sullivan and Atkins, 2009) and chronic myeloid leukemia (Smirnikhina et al., 2016), has important guiding significance for targeted malignant glioma therapy.

**Figure 1:**
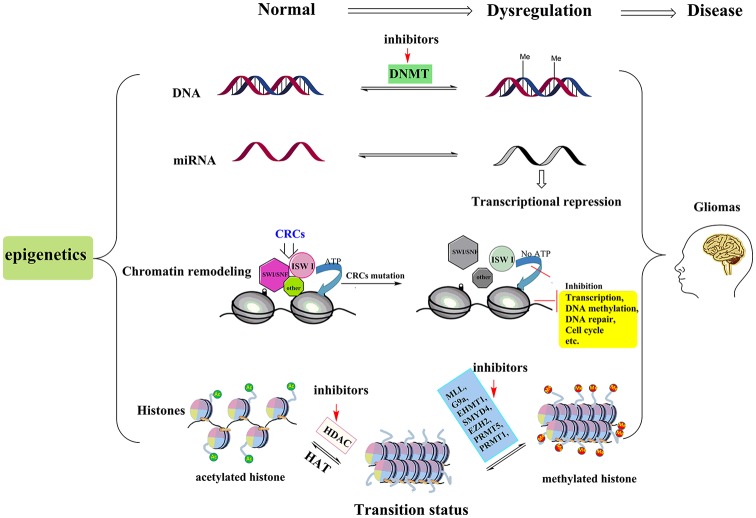
Pathogenesis and treatment options for glioma. The pathogenesis of glioma involves multiple processes. Here we show four epigenetic abnormalities (from top to bottom) linked to the occurrence of glioma: aberrant DNA methylation, abnormal microRNA (miRNA), chromatin remodeling and histone modifications. Among them, chromatin remodeling complexes (CRCs, such as SWI/SNF, ISW I, and other types of complexes) rely on the hydrolysis of ATP to provide energy to complete the chromatin structure changes. When the key proteins of the CSCs are mutated, this leads to abnormalities in the expression of tumor suppressor genes or those genes involved in cell cycle regulation, leading to the occurrence of glioma. In histone modification section, by inhibiting the activities of histone methyltransferases and histone deacetylase (HDAC), more sites in histone tails are free to be acetylated and this process can reverse the aberrant histone modifications, and then further suppress tumor cell proliferation and induce apoptosis. The red arrows represent potential epigenetic-based therapeutic approaches against glioma. For example, in DNA methylation section, DNMT inhibitor, 5-aza-20-deoxycytidine is the representative drug. In histone modifications, the HDACIs (vorinostat, panobinostat, valproic acid, etc.) and relevant histone methyltransferases inhibitors are potential treatment drugs in clinic.

With the advancement of the molecular pathology of malignant glioma, it is now evident that epigenetic abnormalities, including aberrant DNA methylation, abnormal microRNA (miRNA), chromatin remodeling and histone modifications, are closely related to the occurrence of glioma (Kondo et al., 2014; Hashizume, 2017; Figure 1). Multiple enzymes and genes that regulate the epigenetic modifications have become new targets not only for glioma treatments but for the treatment of other cancers as well.

This review summarizes the potential epigenetic-based therapeutic targets and related drugs for glioma. We illustrate how the epigenetic mechanisms dynamically regulate the pathogenesis of the disease. To provide information that will assist clinicians, we discuss preclinical and clinical trials of epigenetic-based treatments of glioma and include the results from these studies. This review also highlights the limitations of the current treatment and suggests future potential areas for research and advancements in the prognosis of the disease.

## DNA Methylation

DNA methylation is one of the earliest discovered epigenetic modifications pathways. There are four possible sites for DNA methylation (Jones, 2012; Lovkvist et al., 2016)—the N-6 position of adenine, the N-4 position of cytosine, the N-7 position of guanine and the C-5 position of cytosine. In mammalian cells, DNA methylation occurs predominantly in cytosine of 5’-CpG-3’ to produce 5-methylcytosine (5mC). The methylation reaction is site-specific and it is performed by the enzyme DNMT with the help of methyl donor s-adenosylmethionine (SAM) as a co-factor (Bird, 2002). Currently, according to the differences in their structure and function, these DNMTs are divided into three categories, DNMT1, DNMT2, DNMT3 (DNMT3a and DNMT3b), with DNMT1 and DNMT3 as the representatives. DNMT1 is involved in the maintenance and extension of methylation, a necessity for non-CpG site methylation. DNMT3a and DNMT3b are *de novo* methyltransferases that methylate CpG. *De novo* methyltransferases may be involved in the regulation of cell growth and differentiation, in which DNMT3b plays an important role in tumor gene methylation (Chédin, 2011). DNMT2 binds to a specific site on DNA and its main target is tRNA (Kaiser et al., 2017). A number of studies (Hashimshony et al., 2003; Krausz et al., 2012; Shimooka et al., 2013) show that DNA methylation can contribute to changes in chromatin structure, DNA conformation, DNA stability, interactions between DNA and proteins, and can also control gene expression. DNA methylation has additionally become an important study of epigenetics and epigenetic genomics due to the close relationship between DNA methylation, human development and tumor diseases, particularly the transcriptional inactivation of tumor suppressor genes caused by methylation of CpG islands content.

### Aberrant DNA Methylation in Glioma

Many studies (de Souza et al., 2018; Liao et al., 2018) have shown that the patterns of DNA methylation in glioma cells are different from those in normal cells. Most notably, the coexistence of extensive hypomethylation and CpG island hypermethylation are characteristic of tumor cells. The DNA methylation status of some relevant genes in glioma is therefore a good biomarker for clinical glioma diagnosis (Figure 1; Qu et al., 2013; Choudhury et al., 2015). Hypermethylation of the gene promoter region is the clearest epigenetic change that occurs in the tumor. The methylation status of the promoter region in the human genome regulates gene expression. Almost all housekeeping genes and roughly half of the tissue-specific genes are under the control of the promoter region. Under normal physiological conditions, most CpG islands are in a hypomethylated state; however, some housekeeping genes, such as DNA repair genes and tumor suppressor genes, are often hypermethylated in tumor tissues (Jin et al., 2017). This aberrant methylation can lead to gene transcription suppression and the loss of their biological function. DNA-5-hydroxymethylcytosine (5hmC), an epigenetic mark resulting from 5mC oxidation, correlates with the progression of glioma (Fernandez et al., 2018). Recent evidence (Kraus et al., 2012) has shown that DNA 5hmC negatively correlates with tumor grade. The CpG island methylator phenotype (G-CIMP) is also used as an indicator of glioma prognosis in infancy, pediatric and adults (Mack et al., 2014; Malta et al., 2018). Importantly, another study (Jha et al., 2014) found that pediatric GBM have a distinct methylome compared with that of adults, suggesting that the G-CIMP indicator of glioma prognosis in adult GBM cannot simply be extrapolated to pediatric GBM and there is a strong need for identification of separate prognostic markers.

Abnormal DNA methylation is an important indicator of the inactivation of tumor suppressor genes. Many tumor suppressor genes in glioma have also been identified, including p16^INK4a^ (Lee et al., 2000), p14ARF (Watanabe et al., 2007), MLH1 (Gömöri et al., 2007) and NDRG2 (Kolodziej et al., 2016; see Table 1). The p16^INK4a^ gene maintains the dephosphorylated activating state of the retinoblastoma tumor suppressor protein (pRb) in the normal cyclinD-Rb pathway to control cell cycle progression. A high frequency (more than 50%) of homozygous p16^INK4a^ gene deletion has been demonstrated in GBM tissues, and p16^INK4a^ is altered in 80% of glioma cell lines. Therefore, restoration of p16^INK4a^ would suppress cell proliferation and induce cell growth arrest (Lee et al., 2000). MGMT is a crucial DNA damage repair gene that can repair alkyl damage caused by BCNU. Esteller et al. (2000) found that MGMT promoter hypermethylation existed in approximately 40% of glioma tissues. The methylation level is related to the occurrence and prognosis of the tumor, which is more important than the prognosis of age and tumor grade (Mur et al., 2015). Besides, the methylation level of MGMT promoter is the most important indicator to assess TMZ sensitivity in glioma treatment, and down-regulated MGMT can substantially restore TMZ chemosensitivity *in vitro* and *in vivo* (Xipell et al., 2016; Yu et al., 2018). Except for the genes mentioned above, the CpG island methylation in gene promoter regions of p73 (Watanabe et al., 2002), LATS1, LATS2 (Jiang et al., 2006), and the genes that are listed in Table 1 are also closely related with the occurrence and development of glioma.

**Table 1:** DNA Methylation in glioma.

Genes/proteins DNA methylation	Location	Pathway	References
p16^INK4a^	9p21	Cell cycle	Lee et al. (2000)
p14ARF	9p21	Cell cycle	Watanabe et al. (2007)
PTPRD	9p23-p24.3	Cell proliferation	Veeriah et al. (2009)
EMP3	19q13.3	Cell proliferation	Alaminos et al. (2005), Kunitz et al. (2007)
KLF4	9q31	Cell proliferation	Nakahara et al. (2010)
P73	1p36.3	Cell cycle and apoptosis	Watanabe et al. (2002)
NDRG2	14q11.2	Cell cycle and proliferation	Kolodziej et al. (2016)
MKP-2	8p12-p11	Cell proliferation	Waha et al. (2010)
NSD1	5q35	Cell proliferation	Berdasco et al. (2009)
miR129-2	11p11.2	Cell proliferation, apoptosis, invasion, and migration	Yadavilli et al. (2015)
HTATIP2	11p15.1	Cell proliferation	Dong et al. (2015)
SLC22A18	11p15.5	Cell proliferation, apoptosis	Chu et al. (2011)
hMLH1	3p21.3	DNA repair	Gömöri et al. (2007)
MGMT	10q26	DNA repair	Weller (2013), Kanemoto et al. (2014), Berghoff et al. (2015)
RANK (TNFRSF11A)	18q22.1	Cell apoptosis	von dem Knesebeck et al. (2012)
Neogenin	15q22.3-q23	Cell apoptosis	Wu et al. (2012)
NAG-1	19p13.11	Cell apoptosis	Kadowaki et al. (2012)
GLIPR1	12q21.2	Cell apoptosis	Li L. et al. (2013)
TES	7q31.2	Cell apoptosis	Bai et al. (2014)
BEX1	Xq22.1	Cell apoptosis	Foltz et al. (2006)
BEX2	Xq22	Cell apoptosis	Foltz et al. (2006)
WNK2	9q22.3	Invasion and migration	Moniz et al. (2013)
AJAP1	1p36.32	Migration	Lin et al. (2012)
CST6	11q13	Invasion	Qiu et al. (2008)
SLIT2	4p15.2	Invasion and migration	Xu et al. (2010)
MiR-124a	8p23.1	Invasion and migration	Fowler et al. (2011)
TFPI-2	7q22	Invasion and migration	Gessler et al. (2011), Vaitkiene et al. (2012)
PCDH10	4q28.3	Cell proliferation, cell cycle progression, and cell migration	Echizen et al. (2014)
RUNX3	1p36	Invasion and migration	Mei et al. (2011)
SOCS3	17q25.3	STAT signal pathway	Martini et al. (2008)
RASSF1A	3p21.3	Ras/STAT signal pathway	Horiguchi et al. (2003), Gao et al. (2004)
RASSF10	11p15.2	Ras signal pathway	Hill et al. (2011)
SFRP1	8p11.21	Wnt signal pathway	Majchrzak-Celinska et al. (2016)
SFRP5	10q24.1	Wnt signal pathway	Götze et al. (2010)
DKK1	10q11.2	Wnt signal pathway	Mueller et al. (2005)
DKK3	11p15.2	Wnt signal pathway	Hara et al. (2015)
NKD1	16q12.1	Wnt signal pathway	Götze et al. (2010)
NKD2	5p15.3	Wnt signal pathway	Götze et al. (2010)
SOX2	3q26.3-27	Migration	Luo et al. (2017)

Isocitrate dehydrogenases 1 (IDH1) is not only a major source of NADPH in the human brain (Bleeker et al., 2010) but also in other body tissues (Kim et al., 2009). The mutations of methylation regulatory proteins IDH1/2 can be detected in most low-grade diffuse astrocytoma (75% mutation rate) and anaplastic astrocytoma (66% mutation rate) as well as oligodendroglioma, mixed promyelocytoma and secondary sex polymorphic neuroblastoma (GBM, 76% mutation rate). Noushmehr et al. (2010) found that the IDH1 mutation is highly correlated with the G-CIMP, and its mutation was associated with the prognosis of secondary GBM and positively correlated with the survival rate of patients (Rossetto et al., 2011). Studies have shown that the hypermethylated phenotype of IDH mutations causes insulator proteins to separate from IDH mutant binding sites (Flavahan et al., 2016). These free insulator proteins are then linked to the normally resting platelet-derived growth factor receptor (PDGFRA) oncogene promoter to form a complex that can further stimulate tumor cell growth. By using demethylated drugs to restore normal function in these insulator proteins, PDGFRA can inhibit the growth of IDH mutant astrocytoma cells (Flavahan et al., 2016). In another study, they associated known subtypes with specific alterations in NF1 and PDGFRA/IDH1 in order to provide a framework for the investigation of targeted therapies (Verhaak et al., 2010). As a critical gene of glioma, IDH has vast potential for the diagnosis, treatment and prognosis of glioma.

## MicroRNA

MiRNAs are short non-coding endogenous RNAs that involve in post-transcriptional gene expression regulation in animals and plants (Bartel, 2004). miRNAs can target complementary regions of the 3’-UTR of the mRNA, inhibiting post-transcriptional processes or degrading mRNA and ultimately reducing protein levels (Pileggi et al., 2013). There are many miRNAs in the human genome, targeting tens of thousands of mRNAs.

### MicroRNAs and Glioma

In recent years, some studies have shown that miRNAs play key roles in the transcriptional regulation and growth and proliferation of various tumor genes (Yan et al., 2017). Therefore, miRNA-based individual therapy and gene editing methods may play important roles in the diagnosis and treatment of glioma. It is currently estimated that about half of the miRNA genes are located in glioma cancer genes or their fragile sites (Table 2), and these miRNA genes can regulate 3% of the entire glioma tumor genes and 30% of the coding genes. Also, a single miRNA can simultaneously affect a hundred mRNAs of GBM (Berindan-Neagoe et al., 2014), whereas a single mRNA of glioma can be modulated by one or more miRNAs (Lakomy et al., 2011).

**Table 2:** Role of microRNA in glioma biology.

MicroRNA					
mi-RNA	Target gene	References	mi-RNA	Target gene	References
**Down-regulation**					
miR-873	GLI1, Bcl-2,	Chen et al. (2015), Zhang J.-S. et al. (2017)	miR-152	Runx2	Zhang P. et al. (2017)
miR-34a	PD-L1	Wang and Wang (2017)	miR-152-3p	NF2	Sun J. et al. (2017)
miR-373	CD44, TGFBR2	Wei F. et al. (2016), Jing et al. (2017)	miR-153	Irs-2	Xu et al. (2011), Ghasemi et al. (2016)
miR-146a	Notch	Hu et al. (2016)	miR-181	VCAM-1, Bcl-2	Chen et al. (2010), Liu et al. (2017)
miR-7	EGFR, PI3K/ATK, Raf/MEK/ERK	Liu X. et al. (2013), Liu et al. (2014)	miR-184	FIH1, SND1	Yuan et al. (2014), Emdad et al. (2015)
miR-128	RhoE	Shang et al. (2016b)	miR-204	IGFBP2	Chen et al. (2016)
miR-195	E2F3, Cyclosporin	Zhang Q.-Q. et al. (2012), Yilaz Susluer et al. (2015)	miR-218	Robo1, E2F2, NF-kappaB	Xia et al. (2013), Zhang et al. (2015), Gu et al. (2016)
miR-124	SCP1, Capn4	Cai et al. (2016), Sun A. G. et al. (2017)	miR-326	SMO	Du et al. (2015)
miR-137	PTP4A3, CSE1L	Li K. K. et al. (2013), Wang et al. (2016)	miR-410	MET	Chen et al. (2012)
miR-15b	Cyclin D1, NRP-2 and MMP-3	Zheng et al. (2013), Sun et al. (2014)	miR-483-5p	ERK1	Wang et al. (2012)
miR-16-1	Zyxin	Li X. et al. (2013)	miR-125b	Connexin43	Jin et al. (2013)
miR-31	radixin	Hua et al. (2012)	miR-138	Immune,	Wei J. et al. (2016)
miR-101	COX-2,	Ma et al. (2016)	miR-145	ABCG2, SOX9, adducin 3	Rani et al. (2013), Shi et al. (2014)
miR-491-5p	EGFR, CDK6 and Bcl-xL	Li et al. (2015)	miR-149	Akt/mTOR, signaling,	Xue et al. (2015)
miR-491-3p	IGFBP2 and CDK6	Li et al. (2015)
**Up-regulation**					
miR-21	Spry2	Kwak et al. (2011)	miR-18a	Neogenin, zonula occluden-1, claudin-5, and occludin	Song et al. (2014), Zhao et al. (2015)
miR-26a	PTEN, TUG1	Huse et al. (2009), Li et al. (2016)	miR-20a	TIMP-2, LRIG1	Wang et al. (2015), Wei et al. (2015)
miR-10b	RHOC	Dong et al. (2012)	miR-23b	TUSC7, TFAM	Jiang et al. (2013), Shang et al. (2016a)
miR-30e	CBL-B	Kwak et al. (2015)	miR-93	integrin-beta8, IL-8,	Fang et al. (2011), Fabbri et al. (2015)
miR-221/222	TIMP2, PTPμ, Cx43, P27Kip1,	Zhang C. et al. (2009), Hao et al. (2012), Quintavalle et al. (2012), Yang et al. (2015)	miR-125b-2	mitochondrial pathway of apoptosis,	Shi et al. (2012)
miR-17-92	CTGF	Ernst et al. (2010)	miR-296-3p	EAG1	Bai et al. (2013)
miR-9/9*	CAMTA1	Schraivogel et al. (2011)	miR-451	SMAD	Gal et al. (2008)

miR-9* is the complementary sequence of miR-9.

miRNAs play many critical roles in the progression of glioma diseases. In particular, miRNAs regulate the expression of cancer-related genes, participate in the regulation of tumorigenesis and regulatory pathways, regulate the differentiation of glioma stem cells, and are encoded by oncolytic viruses and involved in tumor processes. Zhang C. et al. (2012), for instance, found miR-221/222 positively correlated with the degree of glioma infiltration and cell invasion, whereas knockdown of miR-221/222 decreased cell invasion via modulating the levels of the TIMP3 target. Knockdown of miR-221/222 additionally increased TIMP3 expression and considerably inhibited tumor growth in a xenograft model. Another study indicated that the over-expression of miR-221/222 reduced p27kipl levels (Zhang C. et al., 2009). P27kipl prevented cell cycle from G1 to S phase by binding to CDK2 and cyclin E complexes. Therefore, down-regulated miR-221/222 can up-regulate p27kipl to inhibit tumor proliferation.

## Chromatin Remodeling

Chromatin remodeling complexes (CRCs) have ATPase activity and they rely on the hydrolysis of ATP to provide energy to complete the chromatin structure changes (Stanton et al., 2017). Depending on the different subunits that can hydrolyze ATP, the complexes can be divided into SWI/SNF, ISW I and other types of complexes (Figure 1). The SWI/SNF complex and the ISW I complex family were the first to be found in yeast and *Drosophila* (Biegel et al., 2014). The human SWI/SNF complex is a polymer with many molecules, including BRG1, hBMR and tumor suppressor protein Hsnf5, which mainly activates gene transcription and is also involved in the recombination of immunoglobulin and TCR genes (Pulice and Kadoch, 2016). The ISW I complex family include three complexes—RSF, HuCHRAC and CAF1 (Loyola et al., 2003). RSF is a heterodimer that mainly consists of Hsnf-h, which is involved in transcription initiation (Sheu et al., 2010); HucHRAC contains Hsnf-2 h and chromatin assembly factor Hacf1, which is related to the maintenance of the heterochromatin replication status (Hanai et al., 2008); CAF1 is involved in chromatin assembly, altering the state of chromatin to correlate with DNA function (Endo et al., 2017). These complexes and related proteins are associated with activation and inhibition of transcription, DNA methylation, DNA repair and cell cycle.

### Chromatin Remodeling and Glioma

Human diseases caused by abnormal chromatin remodeling are often due to mutations in the key proteins of the remodeling complex. This can lead to the failure of chromatin remodeling in which nucleosomes cannot be correctly positioned, preventing basic transcriptional machinery and the complexes that can repair DNA damage from accessing DNA, which can lead to aberrant gene expression. If these mutations lead to abnormalities of tumor suppressor genes or proteins that regulate cell cycle, they can finally lead to the occurrence of cancer (Marfella et al., 2008; Choi et al., 2015).

Liau et al. (2017) recently indicated that chromatin remodeling regulated GBM drug resistance. GBM stem cells (GSC) can reversibly change to a slow-cycling, long-lasting state when targeted by kinase inhibitors. Under this state, the notch signaling pathway is activated and histone demethylase KDM6A/B is significantly up-regulated. This leads to the removing of trimethylation of H3K27 in genome cis-regulatory region and further leads to the increased levels of H3K27Ac. Chromatin remodeling played a key role in this cellular shift, and this research provided a novel target for the development of effective treatments in the future. By targeting epigenetic and developmental pathways, it is possible to eradicate drug-resistant tumor cells and prevent disease recurrence. Another study (Xiao et al., 2017) revealed evidence demonstrating up-regulated chromatin remodeling factor lymphoid-specific helicase (LSH) promoted the development of glioma. Research (Xiao et al., 2017) indicates that the up-regulated transcription factor E2F1 and glycogen synthase kinase-3β (GSK-3β, an intact complex of E2F1) in astrocytomas and GBM were associated with the progression of glioma and correlated with LSH expression. The depletion of E2F1 decreased LSH expression and cell growth, while inhibition of GSK3β increased the enrichment of E2F1 to the LSH promoter, and increased LSH expression. Lipoprotein receptor-related protein 6 (LRP6), an upstream regulator of GSK3β signaling pathway, was also over expressed in glioma tissue. Knockdown of LRP6 reduced LSH expression level through decreased recruitment of E2F1 to the LSH promoter, finally leading to inhibition of cell growth. Taken together, a mechanistic link between LSH expression and activation of the LPR6/GSK3β/E2F1 axis in glioma illustrates a novel role of LSH in malignant astrocytomas and GBM. Understanding the roles of LSH in glioma progression will not only enrich our knowledge of glioma but also frame LSH as a potential therapeutic target for the treatment of these deadly brain cancers.

## Histone Modifications

In the mammalian epigenome, histone modifications can occur in many ways. The basic unit of histone, called nucleosome, is an octamer that consists of two H2A, two H2B, two H3, two H4 and 147 base pairs wound outside of the composition (de Ruijter et al., 2003; Figure 1). The core histone has C-terminal and N-terminal binding regions. Of these, the N-terminal is particularly relevant, since lysine residue of the N-terminal extends out of the nucleosome and is accessible for modifications, including acetylation, methylation, phosphorylation, ubiquitination and ADP ribosylation. These modifications subsequently alter the expression of the gene without altering the base pair (Wang et al., 2014; Mathias et al., 2015). The total process, known as epigenetic regulation, involves a variety of enzymes. Scientists have classified these enzymes according to their functions (Liu et al., 2011): “writers” (enzymes that add groups such as methyl, acetyl and glycans), “erasers” (enzymes that remove post-translational modifications) and “readers” (enzymes that recognize these epigenetic markers and regulate epigenetic effects). The protein complexes that promote the movement of nucleosomes on chromatin are called “movers.” From this enormous pool, many therapeutic targets are derived as a single target or as combinations, apart from the most prominent DNMTs and histone deacetylases (HDACs).

### Dysregulation of Histone Modifications in Glioma

Aberrant histone modifications can lead to transcriptional abnormalities in gene expression that eventually lead to the development and progression of glioma. Among the various histone modification proteins, two have attracted more attention than the others—HDACs, which cause histone deacetylation, and histone methyltransferases, which cause methylation at various sites of histone (Zang et al., 2014). Among the different classes of HDAC enzymes, HDAC1 (Wang et al., 2017), HDAC2, HDAC3 (Leng et al., 2016), HDAC5 and HDAC9 (Milde et al., 2010) have undergone significant changes in glioma cells. H3 acetylation levels are elevated in high-grade astrocytoma compared to low-grade medulloblastoma and normal brain tissues, as are the expression of HDAC5 and HDAC9 (Milde et al., 2010) in high-grade medulloblastoma. One survey demonstrated that mRNA levels of class II and IV HDACs were down-regulated in GBM compared to low-grade astrocytomas and normal brain tissues (Lucio-Eterovic et al., 2008). The use of HDAC inhibitors (HDACIs) for the treatment of cancer is an area of active investigation. In glioma treatment, HDACIs have been used for the treatment of GBM in combination with RT therapy and chemotherapy (Shi et al., 2016; Ghiaseddin et al., 2018). The anti-tumor mechanism of HDACIs includes blocking cell cycle and promoting cell differentiation, and inducing apoptosis and inhibiting angiogenesis, which can inhibit proliferation and apoptosis of various tumor cells (Marks and Breslow, 2007).

In glioma cells, histone methyltransferases G9a, EZH2, MLL1 and MLL2 (Chang et al., 2009; Cheung et al., 2012; Liu F. et al., 2013; Kondengaden et al., 2016; Wiese et al., 2016; Banasavadi-Siddegowda et al., 2018) regulated the methylation level of lysine located in histone (Table 3); these modifications were closely related to gene transcription regulation and genome integration (Heddleston et al., 2012; Zhou et al., 2016). Protein arginine methyltransferase 5 (PRMT5) is another candidate gene for the diagnosis and treatment of glioma, its nuclear expression correlates with poor survival in glioma patients. Banasavadi-Siddegowda et al. (2018) revealed that GBM cells treated with PRMT5 inhibitor mirrored the effects of PRMT5 knockdown, wherein it led to apoptosis of differentiated GBM cells and drove undifferentiated primary patient-derived GBM cells into a non-replicative senescent state.

**Table 3:** Enzymes and inhibitors related to the pathogenesis of glioma.

Class	Proteins	Inhibitors	References
Histone deacetylases	HDAC2, HDAC9, HDAC1, HDAC3,	Vorinostat, Panobinostat, Romidepsin, Valproic acid,	Lucio-Eterovic et al. (2008)
Histone methyltransferases	MLL, G9a, EHMT1, SMYD4, EZH2, PRMT5, PRMT1	BIX01294, UNC0642, DCG066, EPZ-6438	Chang et al. (2009), Cheung et al. (2012), Liu F. et al. (2013), Kondengaden et al. (2016), Wiese et al. (2016), Banasavadi-Siddegowda et al. (2018)

Inhibiting the activities of histone methyltransferases or HDACs can suppress glioma cell proliferation and induce apoptosis (Sharma et al., 2010; Vargas et al., 2014), suggesting that the inhibitors of these proteins could be candidate drugs for the treatment of glioma. Recently, Ghildiyal and Sen (2017) reported that histone methyltransferase G9a that regulated H3K9 dimethylation has also correlated with the development and progression of glioma, and its inhibitors have also been reported as potential agents for the treatment of glioma (Guo et al., 2016).

It is worth noting that no post-translational modifications processes exist in isolation but rather act with mutual influence and coordination, usually referred to as histone crosstalk. In our earlier research (Zang et al., 2017), we found that simultaneously inhibiting the activity of HDAC and G9a would yield better effects than inhibiting single targets. In our anti-proliferation experiment, multiple cancer phenotypes including leukemia, prostatic carcinoma, hepatocellular carcinoma and pulmonary carcinoma and breast carcinoma were used in this study. Compared to the single target suppression effect, simultaneous inhibition of the activities for two protein targets showed a better anti-proliferation effect in parts of the tumor cell lines, such as breast carcinoma. As illustrated in Figure 1, under the cooperation of G9a inhibitor and HDACIs, more sites in histone tails are free to be acetylated. This acetylation status can activate cancer suppression gene transcription and alleviate the disease. Thus, developing high activity HDAC and G9a hybrid inhibitors is another effective route in targeted epigenetic therapy.

The Polycomb group (PcG) protein family is a group of gene regulatory factors that play a role in embryonic development (Zhao et al., 2017). They are divided into two protein complexes based on function, namely PRC1 and PRC2 (Collinson et al., 2016). PRC2 is a multi-protein complex responsible for the methylation of H3 at lysine 27 (H3K27Me). Zeste Gene Enhancer Homolog 2 (EZH2) is a catalytic subunit that constitutes the PRC2 protein complex (Collinson et al., 2016). Current studies (Orzan et al., 2011; Chen et al., 2017) indicated that EZH2 is over expressed in many tumor tissues, including glioma, and is closely related to the malignant progression, invasion and metastasis of the tumors. In the cellular level of research, EZH2 gene silencing technology or using EZH2 inhibitors prevented glioma cell proliferation (Kurmasheva et al., 2017). Therefore, focusing on EZH2 as a new target may pave a new way for the treatment of clinical glioma (Zhang Y. et al., 2017). In pediatric GBM, H3F3A involves two critical single-point mutations in the histone tail at lysine (K) 27 (K27M) and glycine (G) 34 (G34R/V) that are both involved with key regulatory post-transcriptional modifications (Schwartzentruber et al., 2012). The discovery of these molecular markers provides a basis for the diagnosis of this type of glioma and will lay the foundation for further diagnosis and treatment research.

## Present Clinical Workflow

DNA methylation by DNMT leads to the gene silencing of tumor suppressor genes (Dammann et al., 2017). The inhibition of DNMTs can achieve reactivation of transcription of these critical genes (Castillo-Aguilera et al., 2017). Thus, the study of DNMT inhibitors has become a new bright spot in the treatment of glioma. Among the DNMT inhibitors that have now entered the clinical trials, 5-aza-2′-deoxycytidine (Chu et al., 2013) is the most representative. In tumor cells, 5-aza-2′-deoxycytidine is blended with DNA in the form of phosphate, and then inhibit DNMT activity, eventually leading to the desired low methylation status to exert antitumor effects (Sun et al., 2011). Currently the clinical research of 5-aza-2′-deoxycytidine is limited in leukemia (Roboz et al., 2018) and part of solid tumors (Garrido-Laguna et al., 2013; Fan et al., 2014; lung cancer, etc., not including glioma). For glioma treatment, most of the studies with 5-aza-2′-deoxycytidine are still in pre-clinical research stage (Oi et al., 2009; Zhang et al., 2014).

HDACIs can inhibit glioma oncogene transcription and have a variety of effects on cell life cycle. HDACIs can arrest cell division in G1 and G2 phases, induce cell differentiation and apoptosis (Hazane-Puch et al., 2016), destroy the combination of heat shock protein and substrate protein, promote degradation of oncoprotein, and also inhibit the growth and proliferation of glioma by inhibiting tumor angiogenesis (Pei et al., 2016). DNMT inhibitors and HDACIs can be used for the treatment of various tumors, either individually or as a synergistic combination (Xu et al., 2014; Pathania et al., 2016). In glioma, as a new therapeutic drug, HDACIs provide new directions for the treatment of glioma. Already many HDACIs have entered the phase I/II clinical trials (Table 4) alone or in combination with other chemotherapeutic agents such as TMZ and radiotherapy for the treatment of various types of glioma, including diffuse intrinsic pontine glioma (DIPG), progressive, or recurrent GBM (Lee et al., 2012; Krauze et al., 2015; Kim et al., 2017).

**Table 4:** Summary of epigenetic drugs for glioma that entered in phase I/II clinical trials.

	Clinical trial	Population	Phase	References
Vorinostat	Vorinostat;	Adult: recurrent GBM; Pediatric: refractory solid tumors	II	Galanis et al. (2009), Fouladi et al. (2010)
	Vorinostat and temozolomide;	Pediatric: relapsed or refractory primacy CNS tumors; Adult: high-grade glioma	I	Lee et al. (2012), Hummel et al. (2013)
	Vorinostat and bortezomib;	Pediatric: refractory or recurrent solid tumors; Adult: advanced malignancies	I/II	Muscal et al. (2013), Schelman et al. (2013)
	Vorinostat, erlotinib and radiation;	Adult: GBM (ineffective)	I	Peereboom et al. (2010)
	Vorinostat, temozolomide and radiotherapy;	Adult: GBM	I/II	Galanis et al. (2018)
	Vorinostat and bevacizumab;	Adult: recurrent World Health Organization Grade 4 malignant glioma	II	Ghiaseddin et al. (2018)
	Vorinostat, bevacizumab and temozolomide;	Adult: GBM	I/II	Krauze et al. (2015)
Panobinostat	Panobinostat and bevacizumab;	Adult: recurrent glioblastoma and anaplastic glioma; (no continued accrual)	II	Lee et al. (2015)
	Panobinostat with fractionated stereotactic re-irradiation;	Adult: recurrent HGG	I	Shi et al. (2016)
Romidepsin	Romidepsin;	Adult: recurrent GBM; (ineffective)	I	Iwamoto et al. (2011)
valproic acid	valproic acid;	Pediatric: refractory solid or CNS tumors	I	Su et al. (2011)
	valproic acid, temozolomide and radiotherapy;	Adult: GBM	II	Krauze et al. (2015)

One phase I study conducted by the Children’s Oncology Group (COG) investigated vorinostat with TMZ in relapsed or refractory primary CNS tumors (Hummel et al., 2013). Five-day cycles of vorinostat in combination with TMZ were well tolerated in children with recurrent CNS malignancies, with myelosuppression as the dose-limiting toxicities (DLT). Accumulation of acetylated H3 in peripheral blood mononuclear cells (PBMC) was observed after administration of vorinostat. One phase II trial of vorinostat on recurrent GBM was reported by the North Central Cancer Treatment Group (Galanis et al., 2009). In this study, vorinostat monotherapy was well tolerated in patients with recurrent GBM, and there were obvious increases in acetylation levels of H2B, H3 and H4 after treatment. Microarray RNA analysis showed changes in genes regulated by vorinostat, such as up-regulation of E-cadherin.

With regards to panobinostat, one phase II study of panobinostat in combination with bevacizumab (BEV) was attempted in individuals with recurrent GBM and anaplastic glioma, but part of this study did not meet the criteria for continued accrual and was closed (Lee et al., 2015). Prior to closure, the treatment was reasonably well tolerated in both cohorts, but the addition of panobinostat to BEV did not significantly improve 6-month progression-free survival (PFS6) compared to historical controls of BEV monotherapy in either cohort. More preclinical evidences have shown that panobinostat may act as a radiosensitizer. A phase I trial combining panobinostat with stereotactic re-irradiation in patients with recurrent HGG has been reported (Shi et al., 2016). The results were more promising than panobinostat with BEV, with a PFS6 of 83% in the panobinostat and stereotactic re-irradiation therapy group, compared to 30.4% in the panobinostat with BEV group.

Another phase I study of valproic acid (VPA) in pediatric patients with refractory solid or CNS tumors was conducted by COG (Su et al., 2011). Histone hyperacetylation was observed in half of the patients at steady state. Krauze et al. (2015) recently conducted a phase II study of concurrent radiation therapy, TMZ, and VPA for patients with GBM. The results of this study demonstrated that the addition of VPA to concurrent RT/TMZ in patients with newly diagnosed GBM was well tolerated. VPA may result in improved outcomes compared to historical data and merits further study.

Overall, HDACIs as monotherapy or a combination therapy seem promising in improving prognosis in this difficulty to treat malignancy glioma (Table 4). For the possible toxicity that epigenetic drugs may present during treatment, improving the dosage regimen (Issa et al., 2004) or developing new epigenetic therapies or gaining knowledge of how to synchronize them with other treatment modalities are good choices to alleviate this toxic effect. So far, HDAC inhibitors, vorinostat and valproic acid can both combine with TMZ and/or RT to exert good effects in clinical trials to treat children with refractory or recurrent CNS malignancies or adult GBM (Lee et al., 2012; Hummel et al., 2013; Krauze et al., 2015). This is a good trend for our future clinical research. Vorinostat combined with erlotinib or panobinostat with BEV did not show obvious effects (Peereboom et al., 2010; Lee et al., 2015). Though the results are not optimistic, they provide valuable information for future research. In recent years, immunotherapy combined with other drugs has been a hot topic in cancer treatment, but few clinical trials were reported on combining the HDACIs with Gene-Mediated Cytotoxic Immunotherapy (G-MCI). Most G-MCI were preferred to combine with TMZ and standard of care (SOC) radiation after surgery, and survival outcomes were most notably improved in patients with minimal residual disease after gross total resection (Chiocca et al., 2011; Wheeler et al., 2016).

Although there are few clinical trials on the combination of glioma immunotherapy and epigenetics, many basic studies on immunotherapy and epigenetics of glioma were reported recently (Gallagher et al., 2017; Bhat et al., 2018). Studies have shown that tumor cells can use epigenetic mechanisms to alter their autoimmune origin and disrupt the process of recognition between tumor cells and the immune system. By DNA methylation or histone modifications, tumor cells can directly or indirectly down-regulate the expression levels of key molecules in the tumor immunoreaction process, thereby destroying the immune recognition and killing tumor cells (Maio et al., 2015). At present, the immunotherapeutic drugs that can combine with epigenetic drugs mainly include cytokines immunosuppressive agents, polypeptide vaccines, immunological adjuvants and tumor cell vaccine agents. Epigenetic drugs combined with tumor immunotherapy drugs will become an important research direction for the future treatment of tumors, and also provide new ideas for the treatment of glioma. With the development of improved medical standards, we believe more high-quality phase trials in newly diagnosed and recurrent GBM are imperative.

## Conclusions and Perspectives

Glioma is a common primary malignant brain tumor with high recurrence rates, short survival times, high rates of mortality and treatment difficulties. For all grades of glioma patients, the largest range of safe resection remains the central step in current comprehensive treatment strategies (Kreth et al., 2013). Prior treatments of these tumors had taught us that conventional surgeries and chemo-radiotherapy protocols can only minimally improve the quality of life and slightly prolong the survival of some patients. The postoperative treatments, including radiation, chemotherapy, the dose and the cycle, should be implemented after comprehensive evaluation based on factors such as patient’s age, operation circumstances, histopathological classification and molecular characteristics (Jiang et al., 2016). Further investigations and reviews of the treatment strategies for malignant glioma are needed.

The genetic instability and heterogeneity of glioma are prominent. The mutual regulation mechanisms of related signal transduction pathways, which are not yet fully understood, are essential for the determination of therapeutic targets and drug development process. In the study of epigenetic therapy, multi-target combined inhibition is a critical concept in targeted drug development. In addition to single drug with multi-target inhibition, the combined use of multiple targeted drugs is also important, including those mentioned in this study (e.g., HDAC and G9a). However, even if the targeted drugs are tested in combination, the number of trials is quite large and pre-clinical pre-screening of drugs is therefore necessary. Whether the targeted drug can act on the expected target site and whether it can effectively inhibit the downstream signaling pathways, the potential toxic and side effects, are related to the safety and efficacy of targeted therapy. These issues need to be studied in depth.

Overall, epigenetic modifications are closely related to glioma proliferation, metastasis, invasion and prognosis. Various epigenetic modifications closely interact to participate in the occurrence and progression of glioma. Breakthroughs in the treatment of glioma require advances in scientific research, improvements in therapeutic technologies and protocols, and the development of diagnosis and treatment around individualized protocols. The epigenetic phenomena of glioma, including DNA methylation, abnormal microRNA, chromatin remodeling and histone modifications, have excellent potential significance and application to the diagnosis, treatment, and prognosis of glioma. Among the four fields, the methylation level of the gene promoter region can be taken as a guide for glioma diagnosis, and also related to the prognosis of the glioma (Mur et al., 2015), miRNAs play many critical roles in the progression of glioma (Zhang C. et al., 2012), the protein levels of enzymes that regulate histone modifications are candidates for the diagnosis of the glioma, and its inhibitors are good candidate drugs for the treatment of glioma (Sharma et al., 2010; Vargas et al., 2014). These phenomena might also help monitor high-risk groups, and assist in tumor risk assessment, judgment of tumor recurrence, prediction of tumor treatment efficacy and prognosis and development of specific new target drugs. It is believed that with the improvement of detection methods and experimental methods, promising results will be achieved in the fields of glioma prevention, diagnosis and treatment. Biotherapy, including gene therapy, immunotherapy and targeted molecular therapy, provides a new hope for the treatment of glioma. Some of these therapies have been shown to be effective in preclinical studies and safe in phase I clinical trials (Maio et al., 2015). Yet, clinical trials in phase II and III have been conducted. Based on our review, except HDACIs, a new therapeutic and epigenetic drug that can be taken alone or combined with other drugs or other treatments, the combination of epigenetic drugs with biotherapy is also a particularly interesting and novel direction for the future treatment of tumors. Due to the complicated pathogenesis of glioma, epigenetic applications to glioma clinical treatment are still limited. Discovering more effective therapeutic targets, developing novel targeted drugs, improving the efficacy of existing drugs in clinical research, and reducing the side effects of existing drugs are the problems that we need to face and solve in clinical treatment. With continued research, epigenetics understanding is certain to improve and, so is the epigenetic-based treatment of glioma.

## Author Contributions

XH and LZ conceived and designed the project. Each author has contributed significantly to the submitted work. LZ drafted the manuscript. SK, FC and LW revised the manuscript. All authors read and approved the final manuscript.

## Conflict of Interest Statement

The authors declare that the research was conducted in the absence of any commercial or financial relationships that could be construed as a potential conflict of interest. The reviewer MC and handling editor declared their shared affiliation at the time of the review.
